# How Do Mothers Take Care of Their Infants with Colic Pain? A Mixed-Method Study

**DOI:** 10.4314/ejhs.v31i4.10

**Published:** 2021-07

**Authors:** Behnaz Bagherian, Roghayeh Mehdipour-Rabori, Monirsadat Nematollahi

**Affiliations:** 1 Assistant Professor, Nursing Research Center, Kerman University of Medical Sciences, Kerman, Iran; 2 Assistant Professor, Nursing Research Center, Kerman University of Medical Sciences, Kerman, Iran; 3 Assistant Professor, Nursing Research Center, Kerman University of Medical Sciences, Kerman, Iran

**Keywords:** Infantile Colic, Mothers, Child Care, Infants, mixed-method study

## Abstract

**Background:**

Colic pain is one of the main reasons for stress and anxiety in infants' parents, especially mothers, and there is still no specific treatment. Thus, mothers always try their best to relieve their infants' pain.

The researchers attempted to investigate how mothers take care of their infants with colic.

**Methods:**

This study was conducted with a mixed-method approach and a sequential explanatory design. In the quantitative phase, a cross-sectional study was conducted to assess how to control pain. One hundred fifty mothers of infants with colic living in Kerman, Iran, were chosen by convenience sampling. In the qualitative phase, the researchers interviewed 18 mothers using semi-structured in-depth, and face-to-face approaches. These participants were selected by purposive sampling method. The interviews were analyzed by the conventional content analysis method.

**Results:**

In the quantitative phase, the mothers' most common methods to relieve colic pain were herbal medicines (3.55±0.51) and the change of position (3.35±0.67). The least methods used were probiotics (1.4±0.2) and acupuncture (0). In the qualitative phase, the main theme was “mothers support needs for care,” which includes the following subcategories: “lack of trust in doctor”; “full-time care”; “feeling of inadequacy,” “persistent anxiety, “care without help,” and “looking for ways to control pain.”

**Conclusion:**

Mothers need support to relieve their infants' colic. The provision of educational and psychological supportive packages can be helpful for the mothers. In addition, nurses can help mothers improve their quality of care.

## Introduction

Infantile colic is one of the most common problems in gastroenterology, which leads to frequent complaints and referrals of the infant's family (1). The first definition infantile colic was crying more than three hours a day or crying more than three days a week for at least one month(2). According to the definition of Hymen, colic pain is a constant crying in the evening at a specific time for at least a week in an otherwise healthy child(3). This complication occurs in 10% – 30% of the infants. Its prevalence in Iran is 20% (4).

The pathogenesis of the disease remains unknown. One hypothesis for the cause of colic pain is inflammation of the nervous system and the digestive system of infants. Also, behavioral factors such as family tensions, weak interaction of parents with each other, and the child are discussed. Many risk factors remain unclear, such as an increase in the mother's age and her smoking, which may be related to the extent of colic pain (5). Due to the disease's unknown pathogenesis of the disease, there is no specific treatment to relieve colic pain. Several therapies are also available for treatment. One recommendation is the limited Use of allergens such as cow's milk that may affect pain, and this restriction should be continued for at least two weeks (6). Some studies suggest that nocturnal milk contains melatonin, effectively improves sleep, and reducing colic pain (7). However, the effects of the hydrolyzed probiotic oligosaccharide formula require further studies (8). Some studies have used acupuncture in lowering colic pain, but it is not applied because of the unknown potential side effects (9). Some studies also considered behavioral therapies such as massaging the infant (10).

Baby's constant crying is a primary challenge for child caregivers, especially if resources and support systems for the mother and the child are minimal. If these limitations exist, taking care of the child may be neglected because of the difficulties a mother experiences(11). Infants who cry more may be subjected to physical violence (12). The ability to respond to the needs of the infant depends on the mother's self-esteem and belief in the ability to manage the child. The mother's ability to respond to the needs of the child is the basis of self-efficacy (13).

Lack of self-efficacy causes that mothers do not experience the childcare as a positive event(14). They are seeking effective interventions, but there is still no effective treatment for it (15). According to Waddell (2013), breastfeeding has a protective effect on calming the baby. Furthermore, the correct position of breastfeeding by getting the newborn properly attached to the mother's breast can prevent swallowing air during breastfeeding (11).

Mothers with a colicky baby are at risk for unsuccessful breastfeeding (16). Long and Johns suggested that the parents of these babies were alone and helpless during their infantile colic. According to the research team's experiences, mothers did not have enough mental capacity to take care of their babies properly. Moreover, these mothers were under a lot of stress and anxiety when caring for their colicky babies. Because they are not in contact with nurses, they receive most recommendations and tips from older adults, people around them, and mothers with similar problems. Therefore, they encounter a series of ambiguities and confusions in the care process. Many of their questions regarding managing and controlling colic pain remained unanswered. Quantitative research and questionnaire is not sufficient for understanding the experiences of these mothers in managing their babies' pain, how they address their informational needs regarding pain management, and their strategies in alleviating the baby's pain. Given the limited information available on the experiences of mothers, this study aimed to explain the caring experiences of mothers with a colicky baby.

## Methods

This study was conducted using a mixed-method approach and a sequential explanatory design. The present research was conducted in four medical, educational centres and clinics located in Kerman, Iran, from January 2018 to November 2019. In the quantitative phase, a cross-sectional study was conducted to assess how mothers control colic pain in their babies.

**Samples**: The study population consisted of 150 mothers referred to health and treatment centers affiliated to Kerman University of Medical Sciences. The participants were selected using simple random sampling. The inclusion criteria were as follows full-term infants, absence of known underlying diseases based on the pediatrician diagnosis, the infants' aged 1–6 months, Persian-speaking mothers, and no cognitive disorder.

**Data collection**: For data collection, a pain control questionnaire was developed based on the literature review. This questionnaire had 11 items that measure pain control methods, including behavioral methods, treatment regimen, herbal treatment, and complementary treatment. Each section was scored based on a 4-point Likert scale on the scale value of 1 (never) to 4(always). The questionnaire was distributed among 15 faculty members to ensure content validity, and it was revised according to their comments. In the pilot study, Cronbach's alpha was 85%, indicating questionnaire reliability.

**Study design in the qualitative phase**: In the qualitative phase, conventional content analysis was conducted. The study participants were 18 mothers of infants with colic pain (Table 1). They were selected using a purposive sampling method. The researcher selected information-rich cases related to the phenomenon of interest.

**Table 1:** Demographic characteristics of mothers of infants with colic pai

number of participant	age	level of education	type of job	number of children	economic status
1	28	diploma	Housewife	1	good
2	27	Master's degree	Housewife	2	moderate
3	33	illiterate	Housewife	1	good
4	22	Master's degree	employed	1	good
5	23	Primary education	Housewife	1	moderate
6	20	Master's degree	employed	2	
7	22	diploma	tailor	3	father
8	21	Master's degree	employed	1	moderate
9	25	diploma	Hairdresser	2	moderate
10	27	Master's degree	Housewife	wife1	moderate
11	34	diploma	tailor	3	moderate
12	30	diploma	unemployed	2	poor
13	23	illiterate	Housewife	1	good
14	21	Master's degree	employed	1	good
15	22	Primary education	Housewife	1	moderate
16	23	Master's degree	Housewife	1	poor
17	20	diploma	employed	1	good
18	21	Primary education	worker	2	moderate

**Data collection**: The researcher tried to observe maximum variation in terms of different age groups, socio-economic status, and severity of colic pain. Sampling went on until saturation of information; semi-structured, face-to-face interviews were used for collecting data. A skilled female nurse who was trained for deep interviews (Ph.D. in Nursing) did interviews and she had passed the course on how to interview.

At first, she introduced herself as a nurse and researcher, and then explained the research objectives and the interview process for participants. Then, informed consent was signed.

The appropriate place for interviews was determined by agreement between the researcher and the participant, such as “participants' homes.” The interviews were started with questions such as “how are you affected by the problems of your baby, and” what do you do during infantile colic? The interviews continued using exploratory and probing questions, such as “how did you feel?” “Could you explain more?” etc (Table 2). The interviews lasted from 30 to 90 minutes. The full interviews were recorded with the previous permission of the participants. Also, the key points were written during the interview.

**Table 2:** Guide questions for interview

Questions
How are you affected by the problems of your baby?
What are your problems for caring of your infant?
How do you solve these problems?
What do you do during infantile colic?
How did you feel?

**Data analysis**: In the present study, in the qualitative phase, conventional content analysis was done according to Graneheim and Landman (17). First, the recorded interviews listened for 4–5 times. In the next step, the whole interview was transcribed verbatim in a Microsoft word document. Every transcribed interview was considered as a unit of analysis. Every text was reviewed and corrected by the same interviewee. For better understanding, one of the members of the research team read every finalized text four times, and the meaning units were extracted. Then, the derived meaning units were condensed and coded. The codes were then categorized based on similarities and differences in their meanings. The codes were classified into subcategories according to the degree of relatedness. The interrelations among subcategories were examined, and the main concepts were extracted from them. At the end of each step, the processes used and the findings of that step were discussed among research group members. The final findings were shared with the participants in a meeting, and their final remarks were received. To facilitate the steps of the data analysis process, we used MAXQDA software 10.

**Rigor of study**: The reliability and validity of findings, including credibility, confirmability, dependability, and transferability, were assessed using the criteria proposed by Guba and Lincoln (17). To assure the data reliability, we tried to establish a close relationship and a positive interaction with the participants and encourage them to an extensive collaboration. Moreover, we used the ideas of colleagues, experts, and constant comparisons. We tried to provide the dependability of the findings through constant revisions by experts and participants, and external observers. For data confirmability, we made a great effort to avoid any personal judgment and experiences. For maximum transferability of the results, we tried to explain the data as much as possible.

**Ethical considerations**: Ethical considerations were addressed before the study began. All participants completed written informed consent forms and were assured that their information would remain confidential. This study was approved by grant number 95000612 and the Ethics Committee of Kerman University of Medical Sciences (IR.KMU.REC.1396.1466.).

## Results

**Results in quantitative phase**: The mean age of mothers was 25.4±2.9 years old. Most of them did not have a university degree (68%) and were housewives (59%). The results showed that the most common methods used by mothers to relieve colic pain were herbal medicines (3.55±0.51) and the change in the baby's position (3.35±0.67) and the least used methods were probiotics (1.4±0.2), and acupuncture 0 (Table 2). The chi-square test showed a significant difference between herbal medicines and living in the village (p<0.05). Also, the Use of herbal medicines in extended families was significantly higher (p<0.05) .Younger mothers used significantly more medications to reduce their infants' colic pain (p<0.05) (Table 2).

**Table 2:** Mean and standard deviation Herbal medicine with demographic characteristic in mothers of infants with colic pain

Variables	Herbal Medicine	Statistical test	P-value
Age			
20–25	2.22±0.1	F=2.19	P<.05
26–31	2.45±0.45		
32–36	3.48±0.5		
Residence			
Living in City	2.54±0.03	t= 2.13	P<0.05
Living in Village	3.54±0.23		
Economic Status			
Low	3.06±0.03	F=2.58	P<0.05
Medium	3.48±0.4		
Good	2.8±0.03		
Family status			
Extended Family	3.54±0.04	t=2.17	P<0.05
Nuclear family	2.23±0.5		
Educational status			
primary/middle education and diploma	3.1±1.2	t=-2.15	p>0.05
Bachelor's degree and higher	3.02±1.23		
Job status			
Housewife	3.24±1.2	t=-2.14	p>0.05
employer	3.21±1.4		

**Results in the qualitative phase**: The main theme extracted from data was mothers support needs for care, which includes the following subcategories: “lack of trust in doctor”; “full-time care”, “feeling of inadequacy,” “persistent anxiety, “care without help,” and “looking for ways to control pain” (Table 4).

**Table 4:** The themes extracted the study interviews

Main themes	Themes	subthemes
**support needs for care**	lack of trust on doctor	Frequent visits to the doctor Change doctor
	full-time care	Lack of time for my work Put aside all previous work
	Feeling of inadequacy	Feeling confused Inability to care
	persistent anxiety	Unrest inside Child pain anxiety
	care without help	Feeling lonely Inadequate spouse support
	looking for methods to control pain	Looking for an effective medicine Searching of child pain relief Use of herbal medicines

Mothers' support needs for care were a main them. Almost all of the studied mothers should be supported in educational, psychological, and spiritual domains. The mothers experienced many problems during infantile colic. The mothers did not know what to do to care for their babies. Doctors' different and changing advice and the suspicion of implementing their recommendations were the problems in the care process. Moreover, care without help is another challenge. They were looking for new ways to control their infants' pain, but they were unable to recognize what to do to keep their babies calm.

Lack of trust in the doctor was a subcategory that included two sub-themes “Frequent visits to the doctor” and “Change doctor”.

Most of the mothers believed that doctors were unable to treat the baby, and every time they referred, the doctors changed the medications, so they felt insecure about the doctors. For example, participant 2 said, “whenever I took my child to the doctor, the doctor gave him a new drug; they either changed the medication or wrote an external type. Sometimes, I thought that the doctor could not cure my baby”.

“Full-time care” is another subcategory included sub-themes “Lack of time for my work” and “Put aside all previous work.” The mothers took care of their babies during the colic pain period. Some of them did not have enough time to do their interests. A mother of a three-month-old baby said that she had to do housekeeping when the baby was sleeping because the baby started crying from the evening until the end of the night. Another mother said that she could not do anything at home because she had been involved with her baby.

“Feeling of inadequacy” was the other subcategory that included “Feeling confused” and “Inability to care.”

All mothers became nervous during infantile colic. They could not do anything when their babies had colic. A mother of a 4-month-old baby said that she was living with her parents because the baby started crying at night, and she did not know what to do. She was afraid of her baby's colic pain, and she became restless during infantile colic. Another subcategory was “Persistent anxiety”. Mothers were worried about their babies, even when they were calm. Mother No. 4 said that even when her baby was asleep, she was afraid it woke up with colic pain. Also, “Care without help” was another subcategory, included “Feeling lonely” and “Inadequate spouse support.”

In addition, the main burden of care was on mothers, which reduced their quality of life. Mother No.3 said that “I am bored to take care of this baby, but I must do it.” Mother No. 4 said that she had to take her baby alone because her husband was working two shifts every day.

“Looking methods for pain control” was the last subcategory included “Looking for an effective medicine, “Searching of child pain relief,” and “Use of herbal medicines.”

All of the participants were looking for other methods, including herbal medicines, dietary changes, distraction, and the reduction of environmental stimuli and acupuncture. Mother No. 10 said that she had taken Teucrium Polium so often, according to her mother's experience. However, one of her relatives believed that Kolporeh (Teucrium Polium) would make the baby's skin darker.

## Discussion

In the qualitative phase, we explored the experiences of mothers who were caring for infants with colic pain. The main theme extracted from the data was mothers support needs for care, which includes the following subcategories: “lack of trust in doctor”; “full-time care,” “feeling of inadequacy,” “persistent anxiety, “care without help,” and “looking for ways to control pain.”

Many parents, especially mothers, did not expect colic pain in their babies, and they were not mentally ready to confront this situation. Long-term crying of the infant made mothers anxious and stressed. They need informational, psychological, and spiritual support for caring for their babies.

Daelemans suggests that although this situation is benign, the infant's parents and family are psychologically affected (18). Parents, especially mothers, as the main caregivers of infants, may face many problems during infancy.

Thus, providing accurate information to parents and, in particular, mothers can reduce their uncertainty and help them psychologically. Providing parents with information on how to calm down a baby before dealing with colic pain can help parents manage their situation without consulting people with various sources of information and experiences(19, 20). Having enough information about disease management can improve their self-efficacy and care-giving (19). The findings of studies can be fruitful for parents, especially mothers. Al Saadoon (2018) showed that more than 70% of the families did not know how to control the baby's pain. The Lack of care information has prevented them from taking care of their infants (21).

Regarding the subcategory of the Lack of trust in the doctor, Luyckx K showed that frequent visits with doctors, changes in the treatment and medications were very effective in creating mental problems in the patient and families (22).

Full-time care is another subcategory. In Iranian families, mothers are responsible for housekeeping and taking care of family members, which are difficult for them. Unlike the findings of the current Study, Sabzevari found that employed mothers were more independent, felt more powerful with increased commitment in taking care of children, and they had higher life expectancy (23). In line with the study, Kim extracted a subcategory of “my days for my child.” (24). Besides, another study showed that mothers wanted to develop their relationships with their friends and families (25).

In this study, “mothers' persistent anxiety” is another subcategory. In general, the mother's mental status affects the quality of care. In addition, some studies considered the mother's mental status as an influential factor in determining the severity of the infantile colic. Furthermore, the mother's living conditions and even her job position can affect the infantile colic (21). According to studies, mothers with much stress caused severe infantile colic pain. Mothers are the essential sources of a child. One study recommended psychological support for mothers under anxiety and stress (24).

These findings showed that mothers had a “feeling of inadequacy” in the care process. Owing to the fact that mothers did not correctly understand the pathogenesis of colic pain, its causes, signs, and symptoms, they had difficulty in taking care of their infants.

Moreover, mothers were not aware of the care process, they used errors and trials or the experience of other mothers to take care of their children. One study found that parents felt anxious and unable to take care of child and they had poor self-efficacy (26). Ko found that educational interventions were a key point for increasing the quality of self-care and decreasing anxiety in patients with cancer and their families (25). One study showed that the detailed educational programs for parents were helpful in the improvement of care and control of disease (5). A report on the mothers of children with DM and those with epilepsy manifested a high level of anxiety (27).

In this study, care without a helpline is related to Iranian culture because the mothers had many responsibilities. Their husbands were working outside the house, so they did not have enough time to help their wives. In addition to mothers' reduced quality of care, this issue also affects their quality of life. Howard Sharp reported that caring alone caused depression in mothers of children with cancer in the longrun (28). Thuy showed that family support was related to fatigue of mothers (29). Kim showed that family was one of the power sources for mothers of ill children (24).

In this study, mothers looked for ways to control babies' pain because they tried to calm their children in any way. Many mothers had a positive attitude toward herbal medicines, but they were not certain about them. In this study, the mothers asked different people for different solutions. However, they could not make a definitive decision because of conflicting recommendations.

According to the researcher's experiences, Kermani people commonly use herbal medicines such as Teucrium Polium and peppermint for the alleviation of colic pain.

One of the reasons why mothers have been skeptical about the use of traditional medicines is the unknown pathology of the primary colic pain (30). In addition, various factors, such as mother and baby allergies, mother's type of nutrition, family affective status, and the relationship between mother and father affect the amount and severity of colic pain (21). Today, probiotic drugs are also used to reduce colic pain in infants (31). However, the study mothers used them rarely. In this study, mothers did not apply acupuncture for pain management, but Vaziri F considered medical and herbal drugs, aromatherapy, and acupuncture as treatments of colic pain (32). Some medicines are not recommended for small, low-weight babies because of the possibility of adverse effects (33).

One of the nurses' responsibilities is to use non-pharmacological safe methods, along with certain medications. Some studies reported changing the infants' positions, baby lying on the stomach, and performing abdominal massages as interventions that mothers can take to calm down the infants(34). Nurses should play their role as educators in the community to provide accurate information on how to manage the illness (35). Long-term colic pains cause complications for both families and the baby. It can also lead to child abuse, non-breastfeeding, and an increased prevalence of postpartum depression (21). By providing clear guidelines on the causes and etiology of colic pain and managing such situations, nurses can facilitate this period of life for the infant and the family. Salvatore (2018) showed that strategies such as supporting the family members, ensuring them regarding the child's health, self-limiting nature of the pain, and Lack of long term complications could help parents pass this period successfully (34). This study's limitation study was the mothers were in difficult situations and did not have enough time to interview; it was attempted to interview them appropriately.

The results of this study showed that although we could not prevent and infantile colic pain, nurses should facilitate the care process for mothers with colicky infants. Community and pediatric nurses can help families by informing parents, expressing the physiological nature of the pain, changing family members' viewpoints about child management, and providing proper information on the care of a baby with colic pain. It is also possible to improve the family quality of care by providing mental and educational support packages and writing practical guidelines in this area.
